# Ternary cocktail nanoparticles for sequential chemo-photodynamic therapy

**DOI:** 10.1186/s13046-017-0586-1

**Published:** 2017-09-06

**Authors:** Li Fan, Saisai Zhao, Qian Yang, Jiali Tan, Chaojun Song, Hong Wu

**Affiliations:** 1 Department of Pharmaceutical analysis, School of Pharmacy, and The State Key Laboratory of Cancer Biology (CBSKL), The Fourth Military Medical University, 169th Changle West Road, Xi’an, Shaanxi 710032 China; 2 Institute of Biomedical and Health Engineering, Shenzhen Institutes of Advanced Technology, Chinese Academy of Sciences, Shenzhen, 518055 China; 3 Department of Natural Medicine, The Fourth Military Medical University, 169th Changle West Road, Xi’an, Shaanxi 710032 China; 4 Department of Orthodontics, Guanghua School of Stomatology, Hospital of Stomatology, Sun Yat-sen University & Guangdong Provincial Key Laboratory of Stomatology, Guangzhou, 510055 China; 5 Department of immunology, The Fourth Military Medical University, 169th Changle West Road, Xi’an, Shaanxi 710032 China

**Keywords:** Chemo-photodynamic therapy, Gemcitabine hydrochloride, Docetaxel, Sequential release, Self-decomposable NPs

## Abstract

**Background:**

Previous clinical trials have already demonstrated that combinations of two or more drugs were more effective in the cancer treatment, especially sequential photodynamic design combing with sequential chemotherapy. In our study, we propose a ternary cocktail NP delivery system based on self-decomposable NPs, which could realize synergistic chemo-photodynamic therapy through double loading chemo-drugs and multi-level programmable PDT treatment.

**Methods:**

PS drug methylene blue (MB) was encapsulated into the center of the NP_small_, NP_big&thin_, and NP_big&thick_ carriers through “grown-in” loading mechanism, which was released based on the drug concentration difference of the drug release environment. NP_small_, NP_big&thin_, and NP_big&thick_ carriers have three different drug release profiles, which could realize multi-level programmable PDT treatment. At the same time, antitumor drug gemcitabine hydrochloride (GM) and Docetaxel (DTX), were chosen as the double loading chemo-drugs that absorbed onto the NP_big&thin_ and NP_big&thick_ surface, respectively. In specific, various particle configurations were used for modulating the inner MB sequential release with three pulse T_max_. Also, by adjusting the NP_big&thin_ and NP_big&thick_ configuration, the release interval lag time between absorbed GM and DTX can be successfully modulated to achieve maximized chemotherapeutic efficacy.

**Results:**

In vitro and in vivo results demonstrated that these three pulses T_max_ and the sustained release of MB could maximize the multi-level programmable PDT treatment. And the absorbed GM and DTX also have a release time lag of 12 h, which has been proved as the most effectiveness synergistic interval lag time in the cancer treatment.

**Conclusion:**

Such a precise sequential release manner ternary cocktail NPs provided a promising platform for efficient and safe chemo-photodynamic therapy, which serves as a promising drug delivery system to cure cancer in the future.

**Electronic supplementary material:**

The online version of this article (10.1186/s13046-017-0586-1) contains supplementary material, which is available to authorized users.

## Background

Chemo-photodynamic combination has been manifested great potential to improve efficacy of cancer therapy in recent years. The main aim is to improve therapeutic efficacy at lower drug dose with less toxicity, when compared with single chemo therapy only [1, 2]. Conventional chemo-photodynamic therapy is commonly administrated by simply combining chemotherapeutics with photodynamic therapeutics together [3], however, the temporary synergistic effect still could not maintain the sustainable therapeutic efficacy [4]. The major hole which invalidated the whole work is the tumor relapse, which happened to many cancer treatment period [5].

Tremendous strategies have been devoted to improve the synergistic therapeutic effect [6]. The major combination problem often suffers from different drug pharmacokinetics administration performance, which could not function simultaneously and unify. The emergence of nanotechnology offers an unparalleled opportunity for developing advanced combination drug delivery strategies [7]. Various co-loading drug delivery system (DDS) have been designed using liposomes, polymeric NPs, micelles, and other inorganic NPs [8]. However, co-loading multiple moieties into the same carrier have drawbacks like low drug loading amount, non-factional synchronous, or toxic organic solvents introduce, which could cause severe “efficacy” and “safety” problems. Some researchers tried to adjust precised loading efficacy of two drugs by introducing different chemical bonds, which will cause complicated synthesis routes, toxic organic solvents introducing, low yield and poor reproiducibility. Furthermore, most polymer based nanoparticle (NP) carriers showed remarkable therapeutic efficacy compared to each free drug used solely. However, precise loading and ratiometric delivery of different drugs is still a huge challenge to design NP-based carriers [9–12], and it is hard to adjust the ratio of two different anticancer drugs in these NPs to achieve optimal therapeutic efficacy [13]. Among them, multiple NP cocktails delivery system with sequential photodynamic design has become the most challenge but a widely adopted strategy for long and effective chemo-photodynamic clinical therapy [8]. PDT therapy could realize cell damage through direct and indirect cytotoxicity by reactive oxygen species (ROS) effect, which was produced by light-activated photosensitizer (PS) [14, 15]. Moreover, sustained sequential PDT performance with long duration P-gp inhibition is superior in antitumor efficacy than single PDT treatment [16, 17]. Therefore, the combination of using sequential chemo-drugs cocktails delivery system could largely realize the chemo-photodynamic therapy.

Thus, using three similar NPs for single or doulbe loading PSs and chemotherapeutics with different loading parameters may provide a simple way to realize both the precise drug loading and sequential PDT. In our previous work [18, 19], we have built up NPs systems with adjustable loading efficiency and drug release profiles, so that precise drug loading could be acheived by different NPs. According to the first-line treatment guidances, we could precisely load the drugs onto the NPs surface in a simple way, as well as control the release by adjusting NPs shell thickness and compactness. Also, the PSs could be load in the NPs cores with different parameters to realize sequential release.

**Scheme 1:**
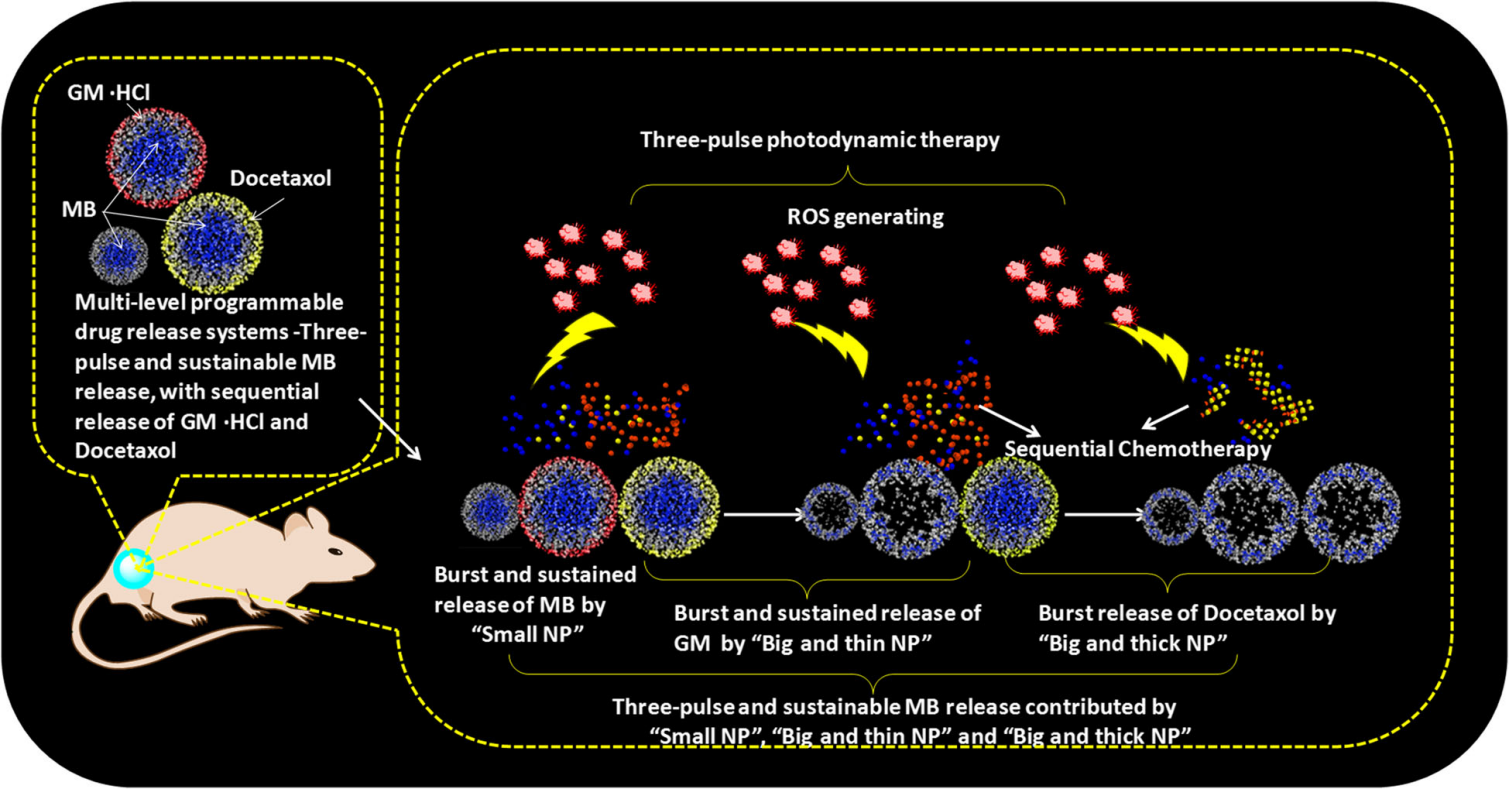
Schematic illustration of multi-level programmable photodynamic system with synergistic sequential chemotherapy

In the present work, we propose a ternary cocktail NP delivery system based on self-decomposable NPs [18, 19]. This NP delivery system could realize synergistic chemo-photodynamic therapy through double loading chemo-drugs and multi-level programmable PDT treatment (Scheme 1). PS drug methylene blue (MB) was encapsulated into the center of the NP_small_, NP_big&thin_, and NP_big&thick_ carriers through “grown-in” loading mechanism, aimed to realize multi-level programmable release of MB for long duration P-gp inhibition. At the same time, antitumor drug gemcitabine hydrochloride (GM) and Docetaxel (DTX), which possess distinct mechanisms of anticancer effect with potent combination regimens in clinics, were chosen as the double loading chemo-drugs that absorbed onto the NP_big&thin_ and NP_big&thick_ surface, respectively. Specifically, NP_small_, NP_big&thin_, and NP_big&thick_ are responsible for modulating the inner grown-in MB sequential release profile with three pulse T_max_ due to the different MB release rates from different particle configurations. In vitro and in vivo results demonstrated that these three pulses T_max_ and the sustained release of MB could maximize the multi-level programmable PDT treatment through P-gp long duration inhibition, thus could maximize the synergistic chemotherapeutic efficacy in the whole process. And the absorbed GM and DTX also have a release interval time lag of 12 h, which has been proved as the most effectiveness synergistic time lag in the cancer treatment [20]. This delivery system will vanish into small pieces (5 nm) due to the self-decomposable NPs configuration. Therefore, such a precise sequential release ternary cocktail NPs provided a promising platform for efficient and safe chemo-photodynamic therapy in the future.

## Methods

### Synthesis and characterization of NP_small_, NP_big&thick_, and NP_big&thin_

The NP_small_, NP_big&thick_, and NP_big&thin_ were synthesized using our pervious synthesis methods [18, 19]. For NP_small_, 2.5 mg of MB was dissolved in ethanol (75 mL) with NH_3_^.^H_2_O, (3.4 mL, 25%, *v*/v), followed by adding 80 μL of Tetraethyl orthosilicate (TEOS). The solution was stirred for 24 h under dark to obtain SiO_2_-MB NP_small_ and washed 4 times by centrifuging at 12000 rpm before freezing dry. For NP_big&thin_ and NP_big&thick_, the protocol was the same with MB amount of 7.5 mg, but different TEOS amount of 80 μL and 120 μL, respectively. Then the dried NP_big&thin_ and NP_big&thick_ was then separately immersed in 1 mL GM^.^HCl (2 mg/mL) and 1 mL DTX (2 mg/mL) to absorb the chemo drug, respectively. NP_cocktail_ was obtained by simply mixed the NP_small_, NP_big&thick_ and NP_big&thin_ at ratio of 1. UV spectrum was conducted to determine the composition of NPs. Meanwhile, NPs were analyzed by TEM (JEM-1400) for particles morphology characterizations.

### Drug release kinetics study in solution

The drug release kinetics of NP_small_, NP_big&thick_, NP_big&thin_ and NP_cocktail_ were determined by UV-vis spectrum. Equal amount of NPs (1 mg/mL) in deionized water were divided into equal groups. Each group was centrifuged at specific time point and the corresponding supernatant was collected. Meanwhile, UV-vis absorption spectra were taken from both re-dispersed particles solution and the supernatants. The morphology evolution of NPs were monitored by TEM.

### Cell culture

Human PDAC cell lines AsPC-1, BxPC-3, MIA PaCa-2 and Panc-1 were kindly provided by Department of immunology, The Fourth Military Medical University. The cells were cultured in RPMI 1640 medium, supplemented with 10% fetal bovine serum, 0.1 g/L streptomycin sulfate and 0.06 g/L penicillin G at 37 °C in a humidified 5% CO_2_ atmosphere. Cell lines were confirmed to be pathogen free.

### Drug release kinetics study in cells

Drug release experiment in vitro was also carried out using Panc-1 cells. Cells were seeded at densities of 5 × 10^6^ cells/mL in 75 mm^2^ flasks and incubated for 24 h, and changed the medium to the one with NPs (NP_small_, NP_big&thick_, NP_big&thin_ and NP_cocktail_, 40 μg/mL). At certain time points, cell samples were collected and washed by PBS for 3 times. After that, the cells were treated with 0.1% Triton X-100 at 37 °C for 5 min, then centrifuged (16,000×g) to separate the released drug molecules from the residual NPs. The drug release in cells were characterized by UV spectrum and the morphological evolution of the NPs were also monitored by TEM.

### Cell viability assay

All of the NPs were sterilized by steaming at 115 °C (NPs in powder form) for 2 h and dispersed in the medium by ultrasonication right before their introduction to the cells. Cells were seeded at initial densities of 5 × 10^4^ cells/mL in dishes and incubated for 24 h before introducing NPs, after that the original NP-free medium were discarded and the fresh prepared NP-containing medium were added. MTT assay was conducted to evaluate the cell viability. Briefly, after incubation with a series concentration of pure drug or NPs for 24 h, cells were transferred to a drug free medium. For each cell line, IC_50_ of GM and DTX was first evaluated respectively. The NPs treated cells were separated for 2 groups, with or without irradiation. The group with irradiation were irradiated with 590 nm LED for series exposure time, then cell samples were further incubated for 24 h. After that, cell culture medium of all samples (free drug, NPs only and NPs with irradiation) were replaced with MTT assay solution (0.5 mg/mL) and the cells were further incubated for 4 h at 37 °C. Then MTT solution was removed and the dimethylsulfoxide was added. The absorbance was measured at 570 nm using amicroplate reader (#680, Bio-Rad). The relative cell viability was calculated as a percentage compared to the control samples (treated with fresh medium without NP/drug). The effect of drug/NPs dose and light irradiation duration to the cell death were checked by MTT assay.

### Quantitative analysis of silica content inside the cells by ICP-MS

For quantitative determination of silica, which was taken up by cells and not attached to the outer cell membrane, cells were washed two times with pre-warmed cell culture medium. Compared with PBS and medium without additives, preliminary tests had shown that full medium at 37 °C can remove extracellular adhered nanoparticles. To remove the contaminative silica, cells were rinsed thoroughly with full medium without detaching cells from the culture surface. Afterward cells were detached with trypsin/EDTA, counted and cell pellets were re-suspended in concentrated hydrochloric acid. Cells were treated with hydrochloric acid and ultrasound for 10 min to get the dissociated silica in the cells. The silica content of the samples was then determined by a photometric assay (Spectroquant®, Merck) and by ICP-MS (iCAP 6000, Thermo Scientific). Experiments were carried out triplicated.

### Assessment of antitumor effect in tumor xenograft

All animal experiments were approved by the Animal Experiment Administration Committee of the Fourth Military Medical University. AsPC-1, BxPC-3, MIA PaCa-2 and Panc-1 cells (5 × 10^6^ cells, total volume 0.1 mL) were injected into mice (Female BALB/C nude mice, 4–6 weeks) leg subcutaneously to establish tumors, respectively. When the diameters of tumors were above 0.2 cm measured by callipers, the mice bearing tumor were randomly divided into 6 groups (5 mice/group) in each tumor model, saline control, free GM, free DTX, Cocktail NPs, Cocktail NPs with irradiation once at 6 h after administration, and Cocktail NPs with irradiation twice at 6 and 18 h, respectively. Free drug or NPs dissolved in saline (with the same drug concentration at IC_50_ level with MB concentration of 10 μM, which were described before, 0.1 mL/mice) were administered through tail intravenous (*i.v.*) injection once a week for 4 weeks. Every mice of Cocktail NPs with irradiation group was conducted light irradiation (590 nm, 1 mW/cm^2^) for 10 min at proper time after injection. All animals were monitored through activity, physical condition, body weight, and tumor growth condition. Bodyweight of each mouse was measured and recorded every week until sacrifice. Before sacrifice, the animals got anesthesia by intraperitoneal injection of pentobarbital sodium (16 mg/mL, 0.1 mL/mice), then tumor masses were removed and weighed. All of the data are reported as the means ± S.D.

### Bio-distribution study

For bio-distribution study, blood samples and tissues were collected 48 h after the final administration. All tissues of one organ were sampled for quantification. For drug concentration of the blood, 1 mL blood were collected from each mice for further study. For tissue NPs concentration determination, anesthesia mice were first infused by saline to remove the remaining NPs in the blood of major organs. Then tissues were weighed and the silica amount were analysis by ICP-MS with the same protocol as the cell ones. In order to make the data comparable directly, all data were normalization processed and present as Mean ± S.D.

## Results and discussion

Ternary cocktail nanoparticles were achieved via combine different configuration self-decomposable NPs, which was named as NP_small_, NP_big&thin_, and NP_big&thick._ There are two drug loading mechanisms based on self-decomposable NPs: one is named as “grown-in”, another is “absorbed”. Light-activated photosensitizer (PS) MB drug was loaded via a co-growth “grown-in” mechanism in both NP_small_, NP_big&thin_, and NP_big&thick_, and anti-tumor drug GM·HCl and DTX absorption onto the big NP_big&thin_ and NP_big&thick_, respectively.

**Fig. 1:**
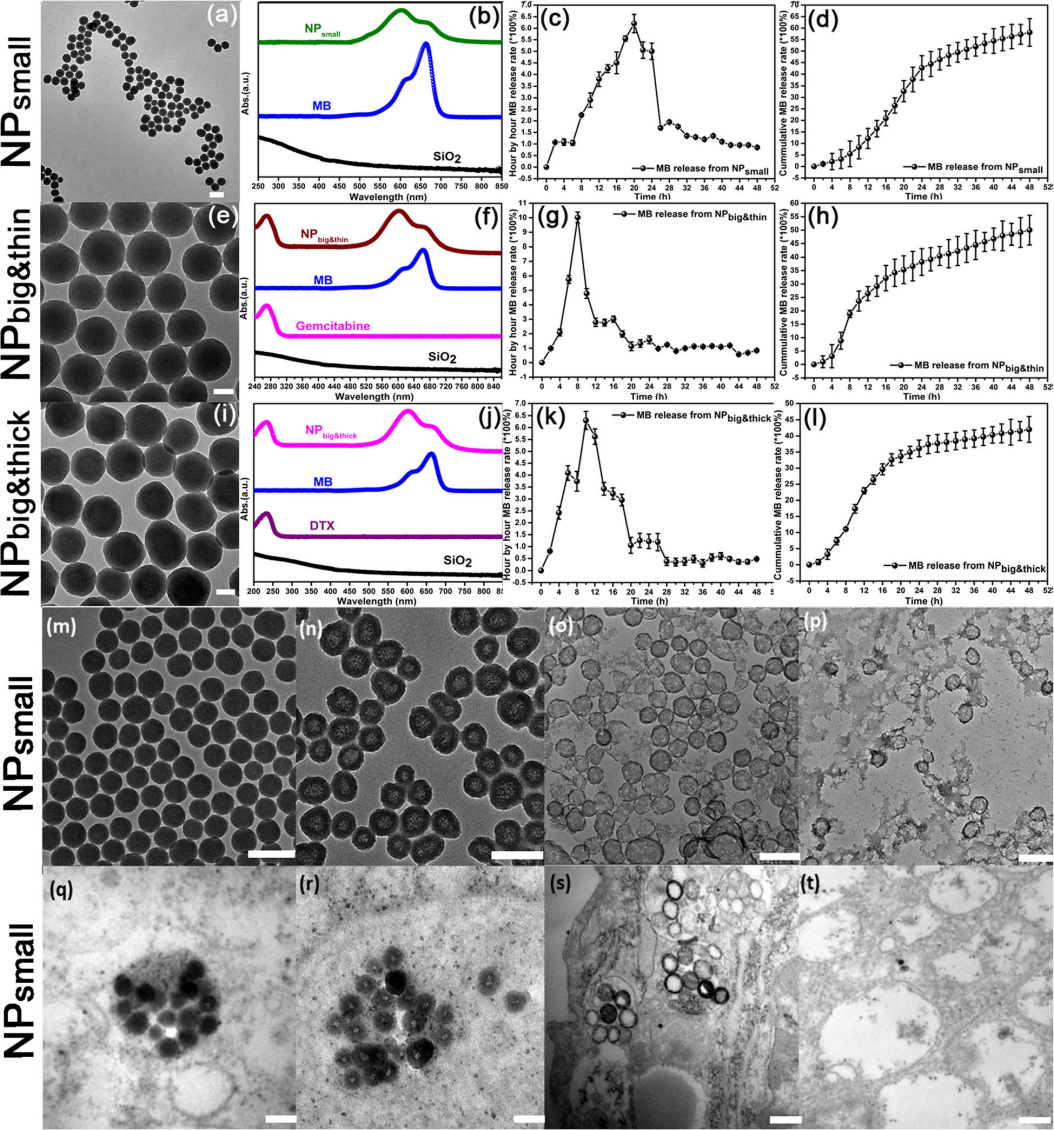
Low magnification transmission electron microscopy (TEM, Philips CM120) image of the synthesized (**a**) NP_small_, (**e**) NP_big&thin,_ and (**i**) NP_big&thick;_ Optical absorption spectra taken from (**b**) NP_small_, (**f**) NP_big&thin,_ and (**j**) NP_big&thick;_ Hour-by-hour MB release profiles of (**c**) NP_small_, (**g**) NP_big&thin,_ and (**k**) NP_big&thick,_ and cumulative MB release profiles of (**d**) NP_small_, (**h**) NP_big&thin,_ and (**l**) NP_big&thick_ from deionized water at 37 °C; Morphological evolution of the NPs by TEM after being immersed in deionized water at 37 °C for (**m**) 1, (**n**) 4, (**o**) 8 and (**p**) 14 days, and Panc-1 cell at 37 °C for (**q**) 12, (**r**) 24, (**s**) 60 and (**t**) 74 h. All scale bars were at 100 nm

The co-growth loading mechanism was realized by introducing MB during the growth of NP_small_, NP_big&thin_, and NP_big&thick_. The synthesis of self-decomposable NP process was mentioned using our tradition methods: introducing 7.5 mg MB-120ul TEOS (NP_big&thick_), 7.5 mg MB-80ul TEOS (NP_big&thin_) and 2.5 mg MB-80ul TEOS (NP_small_) in the synthesis protocol, respectively. In such NPs, MB was most concentrated in the center of the nanoparticle (NP_small_, NP_big&thin_, and NP_big&thick_) with a radial concentration gradient, which could easily falling apart after MB released and leave a center-hollow feature. This feature make the whole NP configuration could self-decompose to 5 nm small piece SiO_2,_ which could easily be excreted from the renal system, avoiding incomplete drug release before carrier excretion [18, 19] and also further proved in Fig. 1m-t. Figure 1a–i shows morphology and size distribution of NP_small_, NP_big&thin_, and NP_big&thick_, which are all spherical in shape (average diameter, NP_small_ ~ 50 nm, NP_big&thin_ ~ 200 nm, and NP_big&thick_ ~ 200 nm) with uniform size and good dispersion. Successful loading of the “gown-in” MB, absorbed GM and DTX into the SiO_2_ NPs were suggested by the optical absorption spectra taken at different stages of the loading process. In Fig. 1c, the characteristic absorption of MB at 660 nm (for monomer) and 600 nm (for dimer) were clearly observed in both NP_small_ but not in the pure SiO_2_ NPs. In Fig. 1f, the absorbed drug GM of NP_big&thin_ gave an obvious optical absorption peak at 270 nm, matching that of pure GM and being absent in the case of pure SiO_2_, suggesting successful loading of GM and MB to the NP_big&thin_. In Fig. 1j, the absorbed drug DTX of NP_big&thick_ gave an obvious optical absorption peak at 230 nm, matching that of pure DTX and being absent in the case of pure SiO_2_, suggesting successful loading of DTX and MB to the NP_big&thick._ Meanwhile, drug encapsulating capabilities have been evaluated using UV-Vis spectrum, following the function below:

Drug encapsulating efficiency = Weight (Drug encapsulation)/Weight (Free drug input) × 100 % .After synthesized, the MB encapsulating efficiency of NP_small_ was ~30%, the MB and GM·HCl encapsulating efficiency of NP_big&thin_ was ~43% and ~96%, and the MB and DTX encapsulating efficiency of NP_big&thick_ was ~47% and ~90%, respectively. Hour-by-hour MB release profiles of NP_small,_ NP_big&thin,_ and NP_big&thick_ from deionized water at 37 °C are shown in Fig. 1c, g, and k, respectively. Optical absorption spectra were recorded from the supernatant to measure the amount of drug release at specific time point. As in the release profile of NP_small_, MB showed a quick rise in the first 8–20 h, followed by a 28 h fade away (Fig. 1c). While the tendency is much faster in NP_big&thin_ and NP_big&thick_, it took only 8 and 10 h to reach the peak (Fig. 1g and k) due to the inner-MB amount and silica shell thickness. Together with the drug release of NP_small_, NP_big&thin_, and NP_big&thick_, which is the whole design methodology of NP_cocktail_, we can get three MB release peaks to realize sequential photo-dynamic therapy first. Such a trend can be clearly seen in the corresponding cumulative profiles of NP_small_, NP_big&thin_, and NP_big&thick_ in Fig. 1d, h, and l, respectively. Furthermore, to get a better mimic of the physiological conditions, the drug release in PBS buffer was also carried out in these three nanoparticles systems. As shown in Additional file 1: Figure S1 in the Supporting Information, similar release tendencies were observed in the PBS buffer. However, due to the salt effect and electrostatic interaction, the drug release in PBS buffer is faster than those in DI water. The differences of release kinetics between DTX and GM∙HCl is due to the large specific surface area and pore capacity of the NPs, the self-decomposable NPs are very easy to absorb small molecule drug onto the NP surface. Furthermore, the large amount of hydroxyl groups in/on the NPs surface, made DTX easier to absorb by intermolecular hydrogen bond. Thus, DTX was absorbed onto the NP surface only by pore adsorption, while GM∙HCl by electrostatic attraction and pore adsorption. So the drug release of GM∙HCl is faster than DTX due to charge force in the release medium, especially in the PBS, culture medium with serum and inside the cells.

**Fig. 2:**
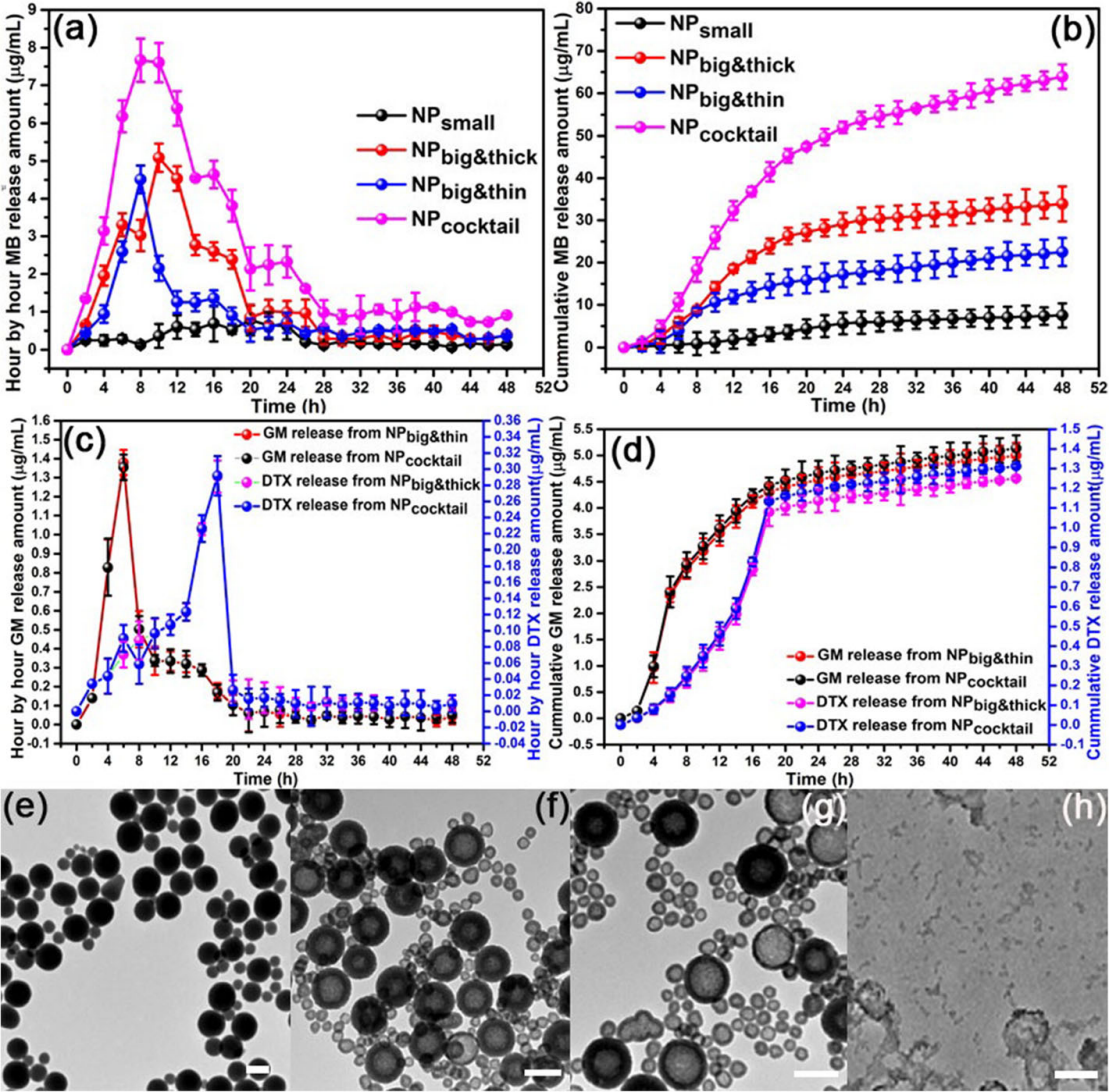
**a** Hour-by-hour and (**b**) cumulative MB release profiles of NP_small_, NP_big&thin,_ NP_big&thick_ and NP_cocktail_ from deionized water at 37 °C; **c** Hour-by-hour and (**d**) Cumulative GM·HCl and DTX release profiles of NP_big&thin,_ NP_big&thick_ and NP_cocktail_ from deionized water at 37 °C; Morphological evolution of the NP_cocktail_ by TEM after being immersed in deionized water at 37 °C for (**e**) 1, (**f**) 4, (**g**) 8 and (**h**) 14 days. All scale bars were at 200 nm

**Fig. 3:**
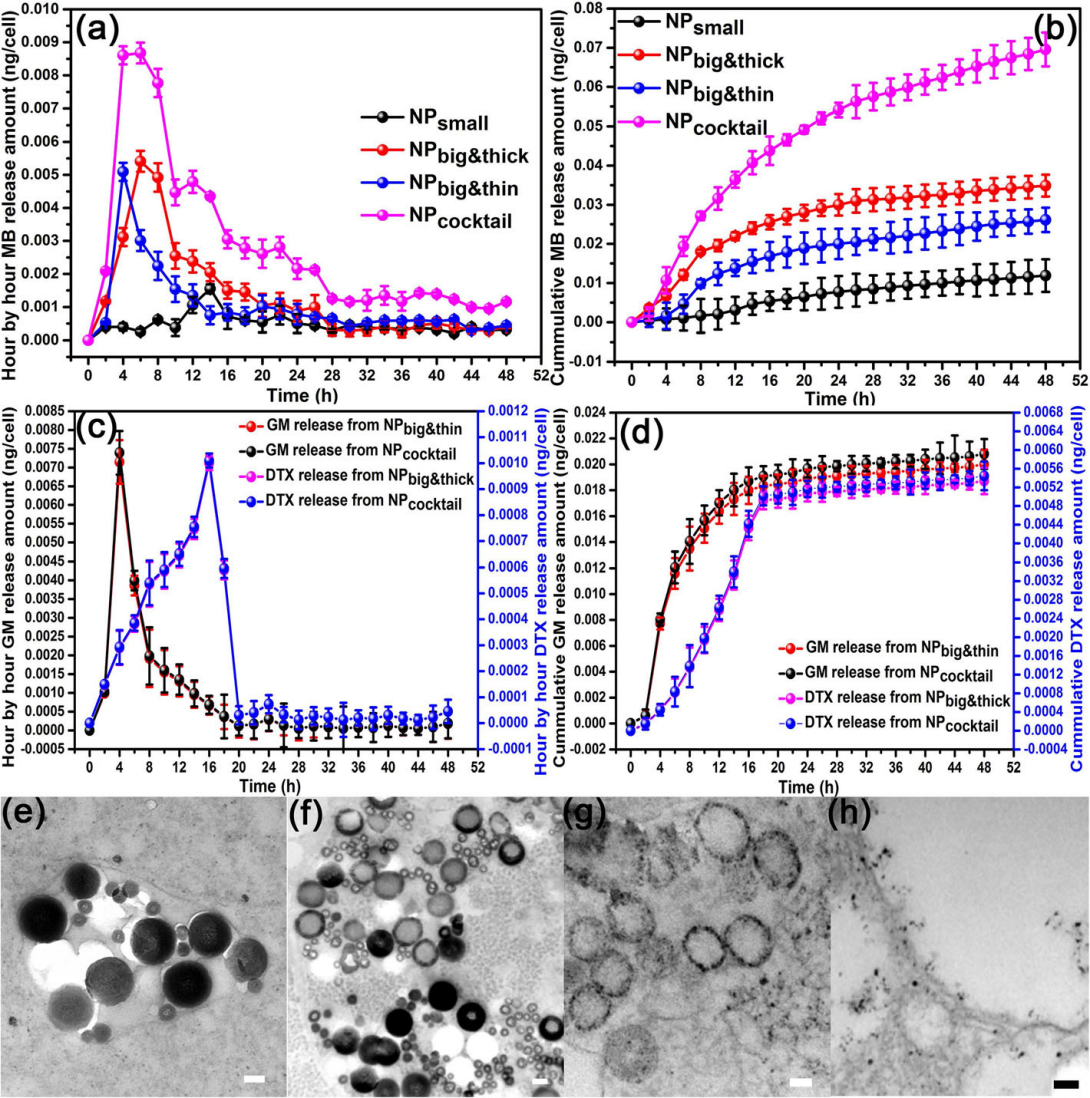
**a** Hour-by-hour and (**b**) cumulative MB release profiles of NP_small_, NP_big&thin,_ NP_big&thick_ and NP_cocktail_ from Panc-1 cell; **c** Hour-by-hour and (**d**) cumulative GM·HCl and DTX release profiles of NP_big&thin,_ NP_big&thick_ and NP_cocktail_ from Panc-1 cell; Morphological evolution of the NP_cocktail_ by TEM after being immersed in Panc-1 cell at 37 °C for (**e**) 12, (**f**) 24, (**g**) 60 and (**h**) 74 h. All scale bars were at 100 nm

**Fig. 4:**
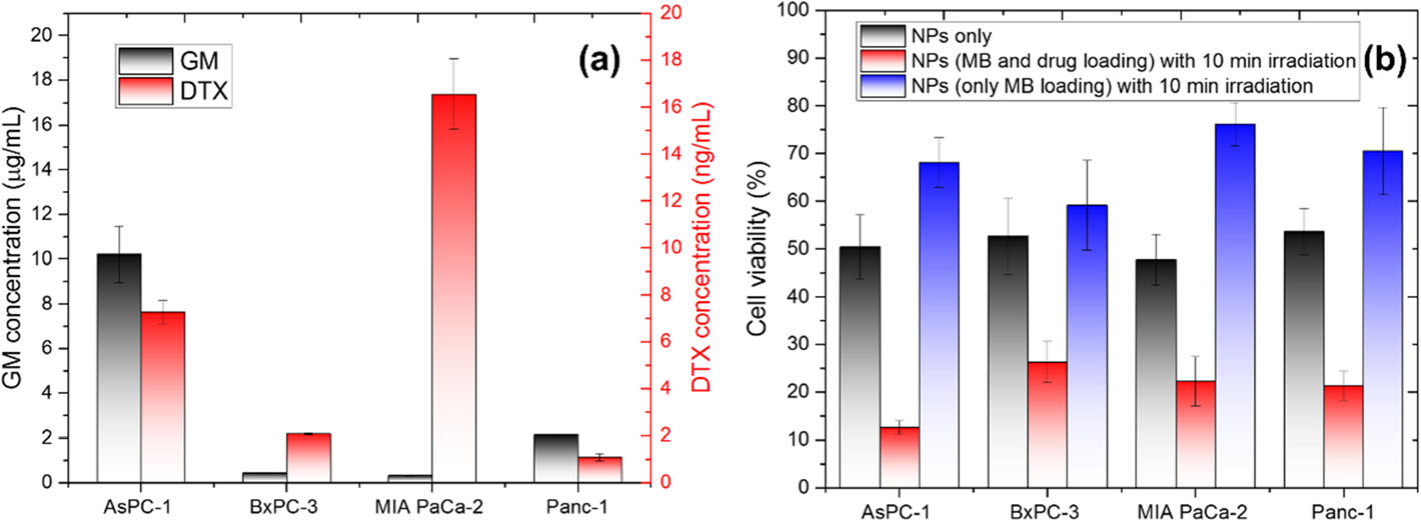
IC_50_ values of (**a**) free GM and DTX treatment groups and (**b**) NPs only without irradiation, MB and drug loading NPs with 10 min irradiation, and only MB loading NPs with 10 min irradiation in 4 different cell lines. Data were presented with mean ± standard deviation (SD) (from 6 independent experiments)

Continuous morphological evolution of the NP_small_ in deionized water at 37 °C were also observed using TEM in the Fig. 1m-t. The whole tendency can be described vividly into four stages: 1) Remained intact-day 1 (Fig. 1m); 2) Shown uniform center-hollow feature-day 4 (Fig. 1n); 3) Nanoshells shown partially damaged-day 8 (Fig. 1o); 4) Vanished into small pieces-day 10 (Fig. 1p). These stages can all be seen from NP_big&thin_ and NP_big&thick_ using different stage time point- NP_big&thin_: 1d-5d-9d-14d and NP_big&thick_: 1d-6d-10d-14d, respectively. Meanwhile, the Continuous morphological evolution of the NP_small_ from Panc-1 cell were also observed using TEM in Fig. 1q-t. The overall morphology change is much faster in the cell than in the H_2_O. The morphology evaluation tendency of different stages matching with much faster real-time point (8 h–12 h-36 h-48 h) due to the faster MB release in lower pH value of endosome. Meanwhile, these stages can all be seen from NP_big&thin_ and NP_big&thick_ using different stage time point-NP_big&thin_: 12 h–18 h-48 h-60 h and NP_big&thick_: 12 h–24 h-60 h-72 h, respectively. Such a characteristic of TEM tendency is matching with the drug release profile, which could well describe the self-decomposable drug delivery system.

Specifically, the NP_cocktail_ combination of NP_small_, NP_big&thin_, and NP_big&thick_ are not only provide MB three peaks and followed sustainable release combination, which was responsible for PDT treatment of the cancer therapy, but also provide chemo drug (GM and DTX) combination, which was responsible for chemo treatment of the cancer therapy. In order to understand NP_cocktail_ chemo-photodynamic therapy behavior, we carried out a number of control experiments in a systematic manner, and studied the release profiles of MB, GM and DTX from all the corresponding NPs at 37 °C deionized water and Panc-1 cell, respectively.

The drug release profiles of inner drug (MB) and outer drugs (GM and DTX) from NP_small_, NP_big&thin_, NP_big&thick_ and NP_cocktail_ have been investigated in water first. The inner drug MB is released due to the inner drug diffusion, which could manipulate by the different NP configurations during the NP synthesis process. Hour-by hour MB release profiles have been observed in water (Fig. 2a). The water release peak from NP_small_, NP_big&thin_, and NP_big&thick_ have been found at 20 h, 8 h, and 10 h, respectively. Therefore, the combination NP_cocktail_ have three relatively peaks appeared at 8 h, 16 h and 24 h, respectively. The overall cumulative release profiles are shown in Fig. 2b. The corresponding absorbed outer drug (GM and DTX) profiles from NP_big&thin_, NP_big&thick_ and NP_cocktail_ have also been investigated in water (Fig. 2c). As for the GM release profile from NP_big&thin_, there is a peak appeared at 6 h due to the outer drug burst release feature. After the burst release, the GM release profile are gradually back to a stable value. As for the DTX release profile from NP_big&thick_, there is a gradually rise tendency first, followed by a burst peak release at 18 h, then back to a stable value. In particular, the burst release peak are 12 h interval time lag between GM and DTX, the main reason is the solubility difference between GM and DTX in water. The corresponding cumulative profiles about GM and DTX are shown in Fig. 2d, which also shown the late DTX burst release feature in the overall profile. The hour-by-hour and cumulative GM release from NP_cocktail_ (Black color) are the same with GM release from NP_big&thin_ (Red color), demonstrating that the existence of NP_small_ and NP_big&thick_ didn’t influence GM release from NP_cocktail,_ and this conclusion also goes to the DTX release from NP_cocktail_ (Blue color) and NP_big&thick_ (Pink color) (Fig. 2c-d). In particular, centrifuged NP_cocktail_ was re-dispersed in DI water at specific time point to study the TEM morphological evolution. As in the hour-by-hour release profile, MB showed three different MB release peaks in the overall profile (Fig. 2a). Together with the drug release, TEM continuous morphological evolution of the NP_cocktail_ also shown at specific time points (Fig. 2e-h). All NPs remained intact at day 1 (Fig. 2e). At day 4, there is hollow feature appeared at all NPs with following hollow degree arrangement: NP_small_ (large) > NP_big&thin_ (medium) > NP_big&thick_ (small) (Fig. 2f), which is agreed to MB peak appeared order in Fig. 2a. Such a uniform center-hollow feature of NP_small_ and NP_big&thin_ continued to enlarge in the following days, leaving a spherical shell with thinner and thinner shell thickness, and began to appear porous. At day 8, some of the nanoshells are even appeared as partially damaged with NP_big&thick_ began to show obviously hollow feature afterwards (Fig. 2g), and all NPs’s nanoshell structure began to collapse into scattered fragments after longer duration (at day 10) (Fig. 2h). TEM continuous morphological evolution of the NP_cocktail_ at 37 °C deionized water also shown three different peak stages in the drug release process.

Meanwhile, the MB release profiles from NP_small_, NP_big&thin_, NP_big&thick_ and NP_cocktail_ in Panc-1 cell are shown in Fig. 3a, which shown release peak from NP_small_, NP_big&thin_, and NP_big&thick_ at 14 h, 4 h, and 6 h, respectively. Therefore, the combination NP_cocktail_ have three relatively peaks appeared at 6 h, 12 h and 22 h, respectively. The overall cumulative release profiles are shown in Fig. 3b. The drug release peak shown in cell is much faster than in water due to the faster MB release in lower pH value of endosome. The three peaks NP_cocktail_ profile from water and cell give us a clearly sequential PS drug profile, which could achieve sustainable PDT treatment in the in vivo experiments. At the same time, GM and DTX profiles from NP_big&thin_, NP_big&thick_ and NP_cocktail_ have also been investigated in Panc-1 cell (Fig. 3c). The drug release peak is much faster in cell compare with water due to the faster drug release in lower pH value of endosome (GM release peak: water-6 h, cell-4 h, DTX release peak: water-18 h, cell-16 h), which also shown 12 h interval time lag in cell. This can also clearly seen from the later DTX burst release feature in the cumulative release profile in Fig. 3d. The hour-by-hour and cumulative GM release from NP_cocktail_ (Black color) are the same with GM release from NP_big&thin_ (Red color), demonstrating that the existence of NP_small_ and NP_big&thick_ didn’t influence GM release from NP_cocktail,_ and this conclusion also goes to the DTX release from NP_cocktail_ (Blue color) and NP_big&thick_ (Pink color) (Fig. 3c-d). Continuous morphological evolution of the combo NP_cocktail_ was also observed using TEM in the Panc-1 cells (Fig. 3e-h). The overall morphology change is much faster in the cell than in the H_2_O. The initial morphology after endocytosis in the cell was shown in Fig. 3a. Then the morphology evaluation tendency from Fig. 3e-h is consistence with Fig. 2e-h, matching with much faster real-time point (12 h–24 h-60 h-72 h) due to the faster MB release in lower pH value of endosome. TEM continuous morphological evolution of the NP_cocktail_ at Panc-1 cell also shown three different peak stages in the drug release process.

Furthermore, for the drug release study performed in in vitro cell cultures, the accumulation of drug molecules in cells may came from two pathways: (1) drug released from nanoparticles that were engulfed by cells and degraded in endosome/lysosome; (2) drug released from nanoparticles that remained in the medium or attached on the surface of cells without being internalized. Both pathways were different from the DI water conditions, and it was expected that the release process which occurred in the endosome/lysosome was faster than the other due to lowered pH. So before fixed the irradiation time in the in vivo study, determination of the endocytosis percentage after specific culture duration is very crucial. As the irradiation time points were set as 6 h and 18 h based on the in vitro results, ICP-MS was employed to determine the silica content after cell exposure to the NPs at these time points. The absolute concentration of silica inside the cells were normalization processed and presented as histogram in Additional file 1: Figure S2. The endocytosis rate at 6 h is over 70% while it reached up to 95% at 18 h (presented as the silica contents). Thus, we could assume that the released MB outer the cells could be neglected and most of the MB inside the cells were attributed to the released MB from NPs inside the endo/lysosome.

It was reported clinical that combination treatment of GM and DTX performed superior antitumor activity when treated with pancreatic ductal adenocarcinoma (PDAC). So in this study, 4 kinds of PDAC cell line were chosen to conduct the in vitro and in vivo study, which are AsPC-1, BxPC-3, MIA PaCa-2 and Panc-1 cell lines. First, cell viability assay was used to evaluate the IC_50_ of GM and DTX respectively in these 4 cell lines. As shown in Additional file 1: Figure S3 (Supporting information), the IC_50_ of free GM drug treatment on AsPC-1, BxPC-3, MIA PaCa-2 and Panc-1 cell line is 10.21, 0.44, 0.32, 2.21 μg/mL, respectively. And the IC_50_ of free DTX drug treatment on AsPC-1, BxPC-3, MIA PaCa-2 and Panc-1 cell line is 7.63, 2.18, 17.38, 1.14 ng/mL, respectively. This IC_50_ value of free drug GM and DTX on different cell lines have been clearly shown on Fig. 4a. Meanwhile, PDT effects comparison of free NP_cocktail_ with 10 mins irradiation on cell viability of different cell lines have been clearly seen from Fig. 4b. The cell viability comparison of NPs without/with irradiation is: AsPC-1 (50%/12%), BxPC-3 (53%/26%), MIA PaCa-2 (49%/24%) and Panc-1 (55%/23%), respectively. Also, the PDT treatment of NP_cocktail_ without loading DTX and GM∙HCl were also carried out for cell viability to demonstrate the MB drug efficiency as a photosensitizer upon irradiation. The results shown that the cell viability in 4 cell lines dropped to 50–70%, suggesting the pure PDT effect in all cell lines. Combined with the chemotherapeutics of the outer drugs GM and DTX, NP_cocktail_ showing significant chemo-PDT effect in the whole cancer treatment.

In order to avoid the influence of NPs on the cytotoxicity, blank NP_small_, NP_big&thin_ and NP_big&thick_ were also evaluated using MTT assay (Additional file 1: Figure S4). The NPs of all parameters present no cytotoxicity even in high concentrations in 4 PDAC cell lines. Safety range time of irradiation was also evaluated using irradiation alone at various exposure time. Cells in 4 cell lines were safe upon irradiation for 10 min under 590 nm LED (Additional file 1: Figure S5a). In order to provide the study implementation in vivo experiment of antitumor efficacy, IC_50_ concentrations of both drug in 4 cell lines was used as a guideline for preparation of nanoparticles at proper GM/DTX ratio. For AsPC-1 cell line, GM onto NP_big&thin_ was used equal amount as DTX onto NP_big&thick_ (GM_NPbig&thin_ /DTX_NPbig&thick_ = 1). Similarly, for BxPC-3 cell line, GM_NPbig&thin_ /DTX_NPbig&thick_ = 200; for MIA PaCa-2 cell line, GM_NPbig&thin_ /DTX_NPbig&thick_ = 20; for Panc-1 cell line, GM_NPbig&thin_ /DTX_NPbig&thick_ = 2000. MB amount in all NP_big_ was kept the same (10 μM, 32 μg/mL). MTT assay were then carried out at 4 cell lines with feeding respective GM/DTX ratio NPs at IC_50_ concentration with light irradiation for 0, 5, 10 mins. As shown in Fig. 4b and Additional file 1: Figure S5b, at fixed concentration of MB and ratio of GM/DTX around IC_50_, cell viability of 4 cell lines all reduced to half after 10 min irradiation. Thus, the administration parameter of in vivo test was set as NPs dose at IC_50_ value with 10 min irradiation 24 h followed.

**Fig. 5:**
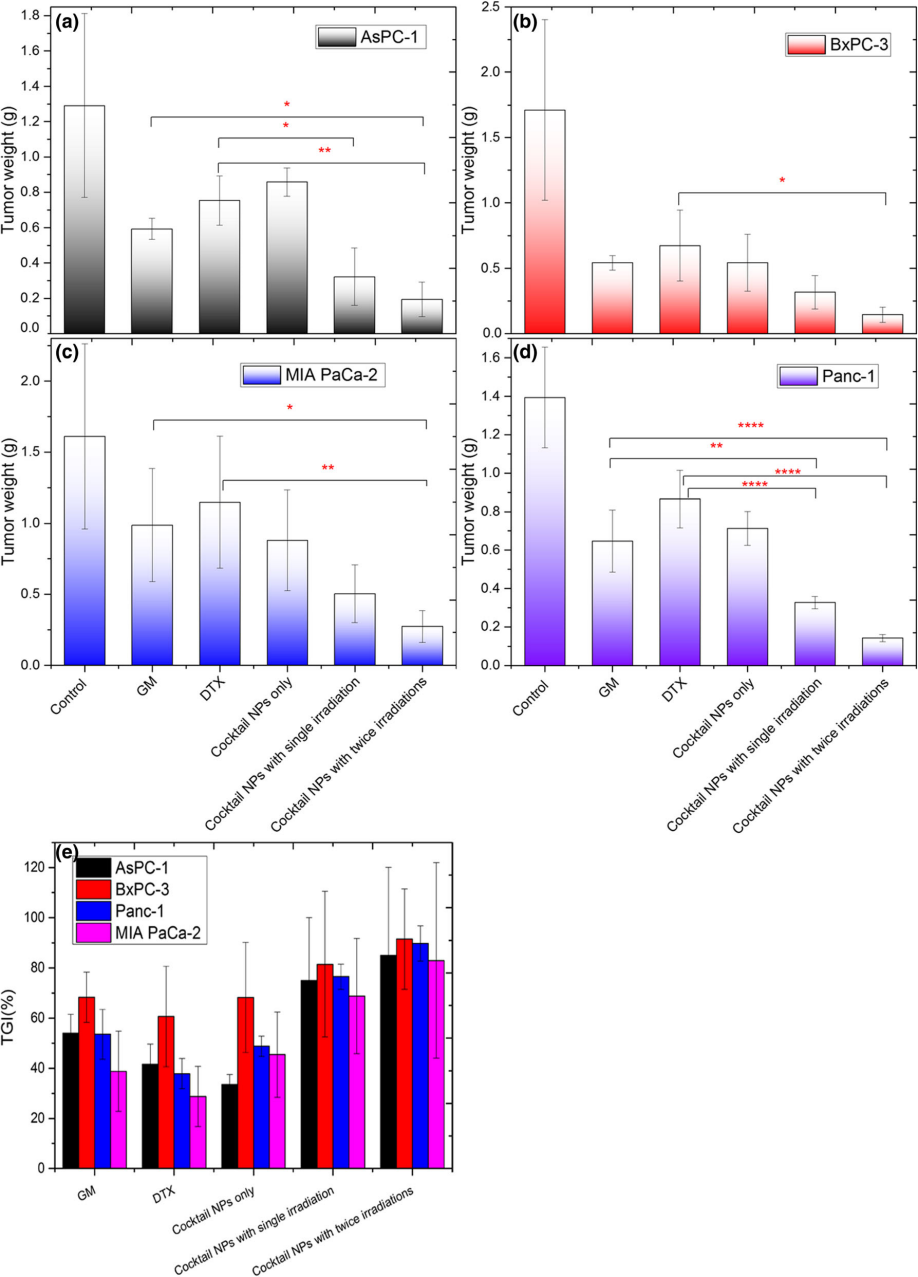
Antitumor effect of Cocktail NPs on nude mice bearing (**a**) AsPC-1, (**b**) BxPC-3, (**c**) MIA PaCa-2 and (**d**) Panc-1 cells subcutaneously was studied. **e** The tumor growth inhibition (TGI) rate was also used to quantitatively measure the tumor suppression in all 4 xenograft tumor models. Values of tumor weight are expressed as mean ± SD (*n* = 5). Data are presented as from 5 independent experiments and significantly different *P* < 0.05 (*), *P* < 0.01 (**), and *P* < 0.0001 (****) from free drug treatment group (analyzed by Sidak’s multiple comparisons test)

In vivo antitumor assessment was evaluated in xenograft mice models by intravenous (i.v.) injection. The Cocktail NPs with twice irradiations at peak release time (6 h and 18 h) respectively significantly delayed subcutaneous tumor growth in all 4 tumor models, as demonstrated by the tumor weight evolution (Fig. 5 **,** Additional file 1: Table S1-S4 and Figure S6). The tumor growth inhibition (TGI) rate was also used to quantitatively measure the tumor suppression (Fig. 5e). The TGI was calculated on the day we finished the experiment, with the formula below, TGI(%) = (W_c_-W_t_)/W_c_*100, where W_c_, W_t_ are the median weight of control and treated groups at the end of the study. In the absence of light irradiation, no significant differences in the efficacy of Cocktail NPs were observed in all tumor models when compared with free GM or DTX. Futhermore, only in AsPc-1 and Panc-1 tumor models, significant tumor growth delay could be observed in Cocktail NPs with single irradiation when compared with free drugs (Fig. 5a, d and Additional file 1: Table S1 and S4). Nevertheless, significantly delayed tumor growth was achieved in the Cocktail NPs with twice irradiations in all 4 tumor models (TGI% > 80%), as compared to that treated by control, free GM and DTX, revealing the notable PDT antitumor efficacy (Fig. 5 **,** Additional file 1: Table S1-S4). The results are consistent with the in vitro MTT findings. The bio-distribution of the cocktail NP systems were quantitative analysis by ICP-MS, and silica amount were presented for the NPs in the major organs. As shown in Additional file 1: Figure S7, we could see that the nanoparticles systems could be easily scavenged by RES system and tend to accumulate in liver in a significant proportion (about 50%, mainly due to the large size of NP_big_ ones), and the left half ones will accumulate in tumors due to the EPR effect.

**Fig. 6:**
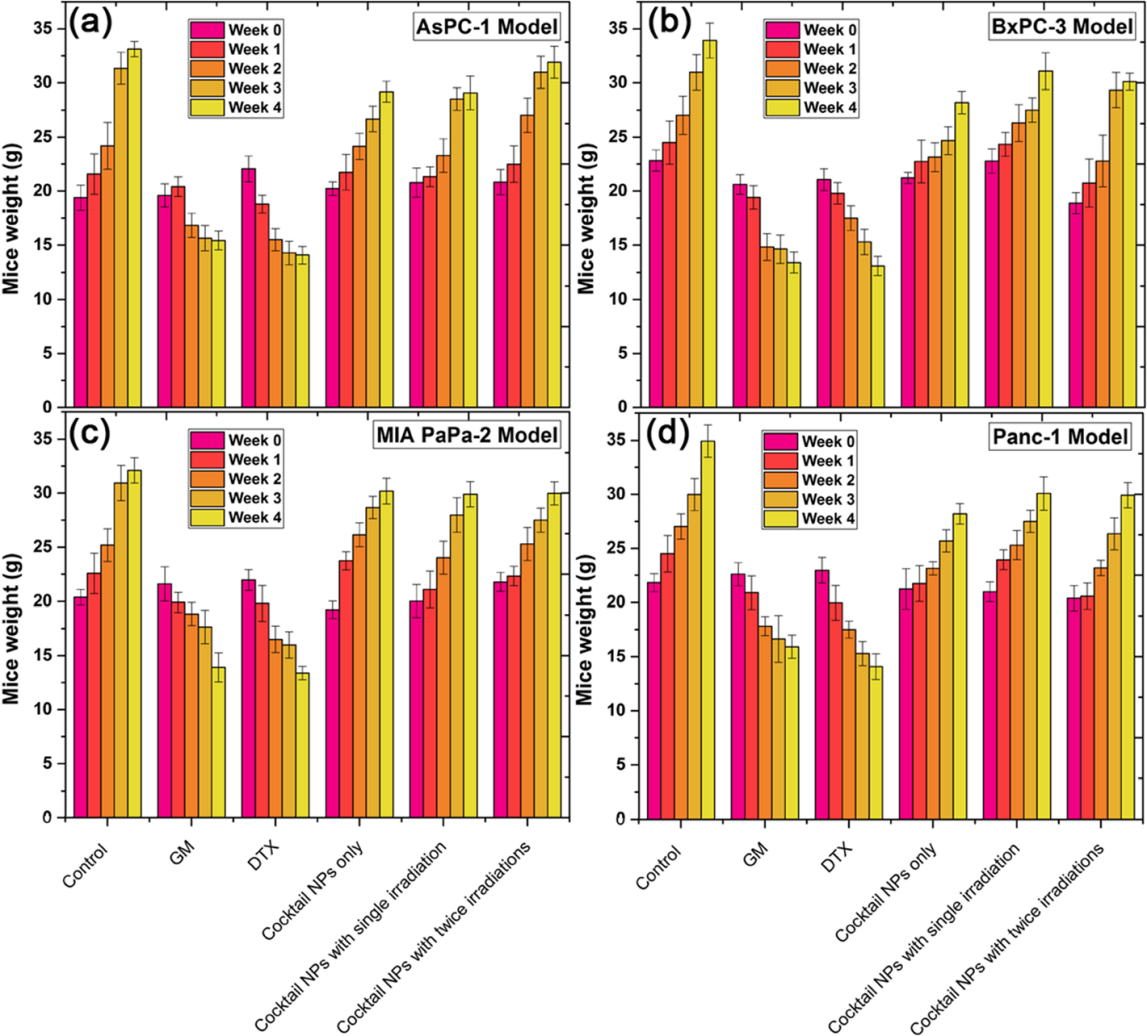
The effects of Cocktail NPs treatment on nude mice bearing 4 different PADC cells. Values of body weight changes in (**a**) AsPC-1, (**b**) BxPC-3, (**c**) MIA PaCa-2 and (**d**) Panc-1 cells bearing mice are expressed as mean ± SD (*n* = 5). Mice were administered via *i.v.* injection every 5 days for 4 weeks

Furthermore, changes in body weights were also investigated to preliminary evaluate the systemic toxicity of NPs treatment groups in all 4 tumor models. The results can be found in Fig. 6. Comparing to the control groups, free drug treatments significantly reduced the body weight of mice after four weeks, indicating potential toxicity of free drug in the applied dosage regimen in all 4 animal models. While the normal body weight increase of the NPs treated groups with/without irradiation were always comparable to that of control groups (Fig. 6). The results demonstrated that using cocktail NPs as delivery systems could be able to reduce the potential systemic toxicity of GM and DTX.

## Conclusion

In conclusion, we have designed a cocktail NPs chemo-photodynamic combination cancer treatment system based on our self-decomposable NPs. The programmable inner drug MB have been well designed to achieve three peaks release, which could maximum the PDT effect of the treatment. Meanwhile, the outer drug GM and DTX have been well designed to achieve 12 h interval time lag, which could maximum the chemo therapeutic effect. The present cocktail nanoparticle configuration and the loading/maintenance release mechanisms provide a promising platform that ensures a sequential chemo-photodynamic therapy, which serves as a promising drug delivery system to cure cancer in the future.

## Additional file

Additional file 1: Figure S1. Cumulative MB release of (a) NPsmall, (c) NPbig&thin, and (e) NPbig&thick and hour-by-hour MB release profiles of (b) NPsmall, (d) NPbig&thin, and (f) NPbig&thick, from PBS buffer at 37 °C. **Figure S2.** Silica content of Panc-1 cells presented in percentages after 6, 18, and 24 hrs incubation with cocktail nanoparticles. **Figure S3.** IC50 determination of free GM and DTX treatment groups in 4 different cell lines. **Figure S4.** Cytotoxicity evaluation of NPsmall, NPbig&thin and NPbig&thick in 4 different cells. **Figure S5.** Cytotoxicity evaluation of Cocktail NPs. **Figure S6.** The tumor size growth curves during the treatment in (a) AsPC-1 cells, (b) BxPC-3 cells, (c) MIA PaCa-2 cells and (d) Panc-1 cells. **Figure S7.** The bio-distribution of cocktail NPs presented by Silica percentage in (a) AsPC-1, (b) Panc-1, (c) MIA PaCa-2 and (d) BxPC-3 tumors bearing mice. **Table S1-S4.** Statistical analysis of tumor weight treated with Cocktail NPs upon different irradiation protocols in AsPC-1, BxPC-3, MIA PaCa-2 and Panc-1 tumor model. (DOCX 1397 kb)

## Abbreviations

DDS: Drug delivery system
DTX: Docetaxel
GM: Gemcitabine hydrochloride
MB: Methylene blue
MTT: 3-[4, 5-dimethylthiazol-2-yl]-2,5-diphenyltetrazolium bromide
NP: Nanoparticle
PBS: phosphate buffered saline
PS: Photosensitizer
ROS: Reactive oxygen species
TEM: Transmission electron microscopy
TEOS: Tetraethyl orthosilicate
