# Supplementary material for: Ternary cocktail nanoparticles for sequential chemo-photodynamic therapy
Source: J Exp Clin Cancer Res. 2017 Sep 6;36:119. doi: 10.1186/s13046-017-0586-1 (PMC5585930; doi:10.1186/s13046-017-0586-1)
Supplement: Additional file 1: Figure S1. — Cumulative MB release of (a) NPsmall, (c) NPbig&thin, and (e) NPbig&thick and hour-by-hour MB release profiles of (b) NPsmall, (d) NPbig&thin, and (f) NPbig&thick, from PBS buffer at 37 °C. Figure S2. Silica content of Panc-1 cells presented in percentages after 6, 18, and 24 hrs incubation with cocktail nanoparticles. Figure S3. IC50 determination of free GM and DTX treatment groups in 4 different cell lines. Figure S4. Cytotoxicity evaluation of NPsmall, NPbig&thin and NPbig&thick in 4 different cells. Figure S5. Cytotoxicity evaluation of Cocktail NPs. Figure S6. The tumor size growth curves during the treatment in (a) AsPC-1 cells, (b) BxPC-3 cells, (c) MIA PaCa-2 cells and (d) Panc-1 cells. Figure S7. The bio-distribution of cocktail NPs presented by Silica percentage in (a) AsPC-1, (b) Panc-1, (c) MIA PaCa-2 and (d) BxPC-3 tumors bearing mice. Table S1-S4. Statistical analysis of tumor weight treated with Cocktail NPs upon different irradiation protocols in AsPC-1, BxPC-3, MIA PaCa-2 and Panc-1 tumor model. (DOCX 1397 kb) [file 13046_2017_586_MOESM1_ESM.docx]

Additional file

Ternary cocktail nanoparticles for sequential chemo-photodynamic therapy

Li Fan^a1^, Saisai Zhao^b1^*, Qian Yang^c^, Jiali Tan^d^, Chaojun Song^e^, Hong Wu^a^*

^a^ Department of Pharmaceutical analysis, The Fourth Military Medical University, 169th Changle West Road, Xi’an, Shaanxi, China, 710032.

^b^ Institute of Biomedical and Health Engineering, ShenZhen Institutes of Advanced Technology, Chinese Academy of Sciences, Shenzhen, China, 518055.

^c^ Department of Natural Medicine, The Fourth Military Medical University, 169th Changle West Road, Xi’an, Shaanxi, China, 710032.

^D^ Department of Orthodontics, Guanghua School of Stomatology, Hospital of Stomatology, Sun Yat-sen University & Guangdong Provincial Key Laboratory of Stomatology, Guangzhou, China, 510055.

^e^ Department of immunology, The Fourth Military Medical University, 169th Changle West Road, Xi’an, Shaanxi, China, 710032.

* Correspondence to S. Zhao (ss.zhao@siat.ac.cn) and H. Wu (wuhong@fmmu.edu.cn)

^1^ These authors have equally contributed.


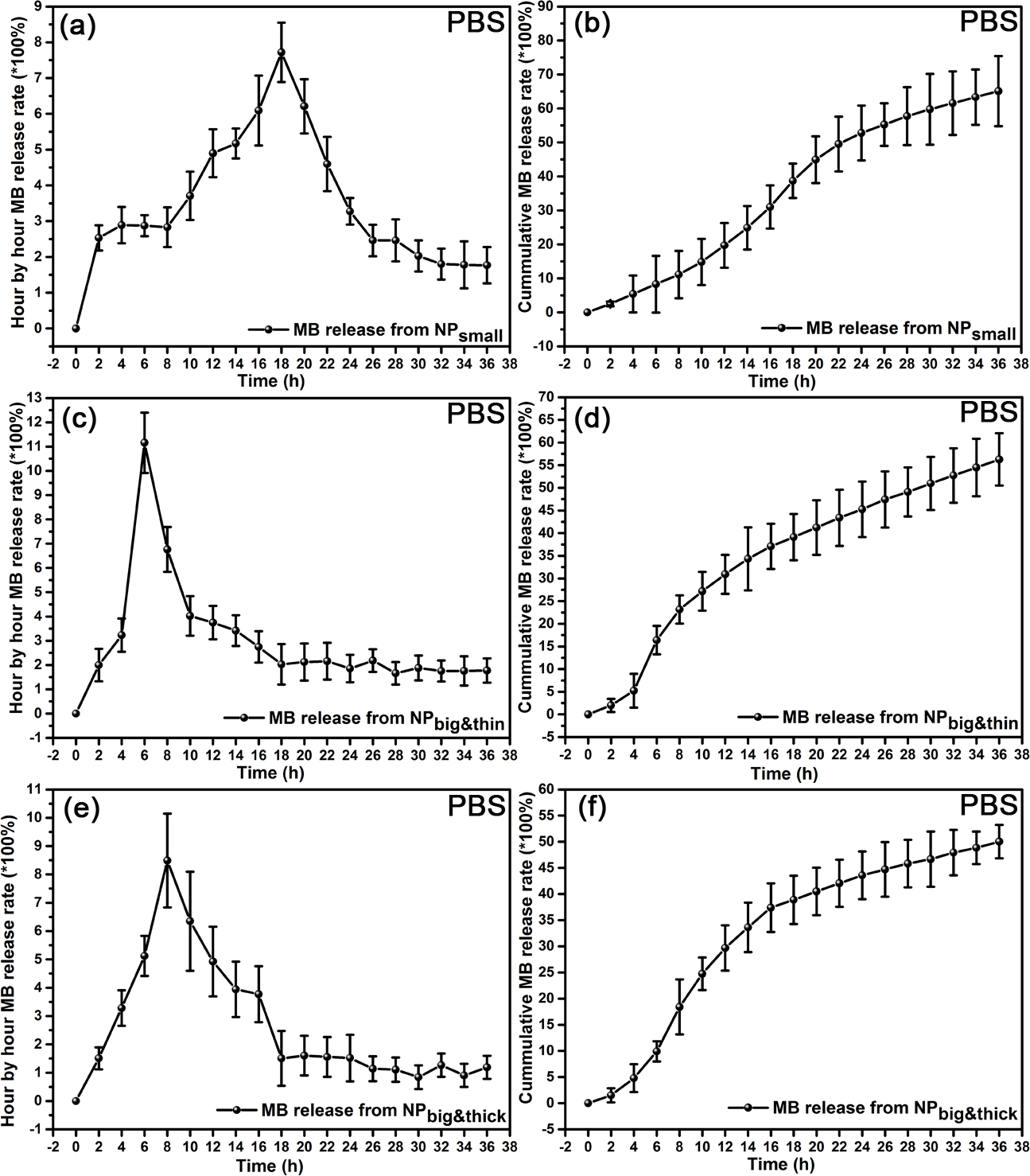


**Figure S1** Cumulative MB release of (a) NP_small_, (c) NP_big&thin,_ and (e) NP_big&thick_ and hour-by-hour MB release profiles of (b) NP_small_, (d) NP_big&thin,_ and (f) NP_big&thick,_ from PBS buffer at 37 °C. All experiments were carried out triplicate and the data was shown as Mean±S.D.


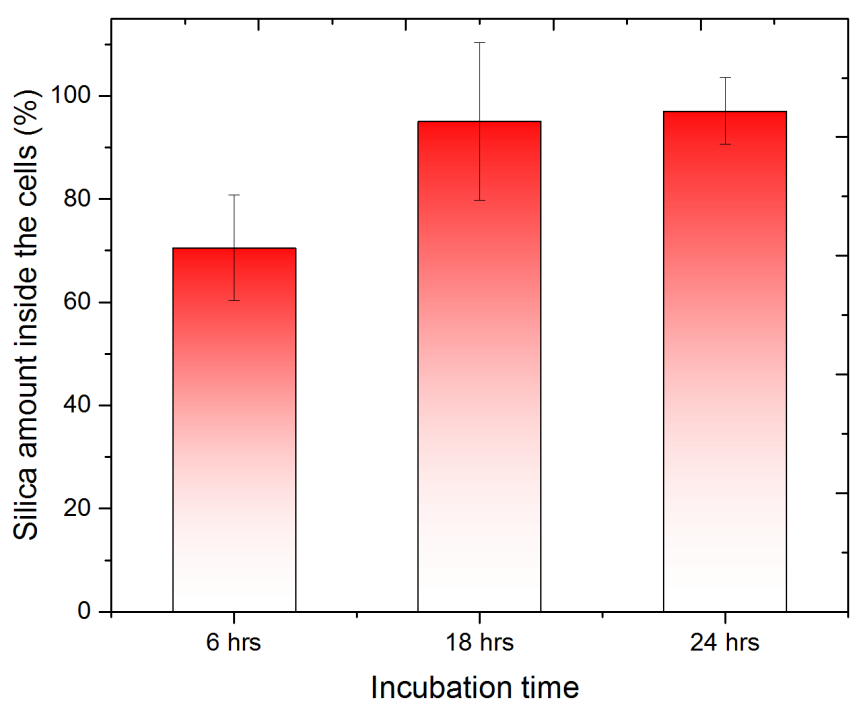


**Figure S2** Silica content of Panc-1 cells presented in percentages after 6, 18, and 24 hrs incubation with cocktail nanoparticles. All experiments were carried out triplicated and the data were shown as Mean±S.D.


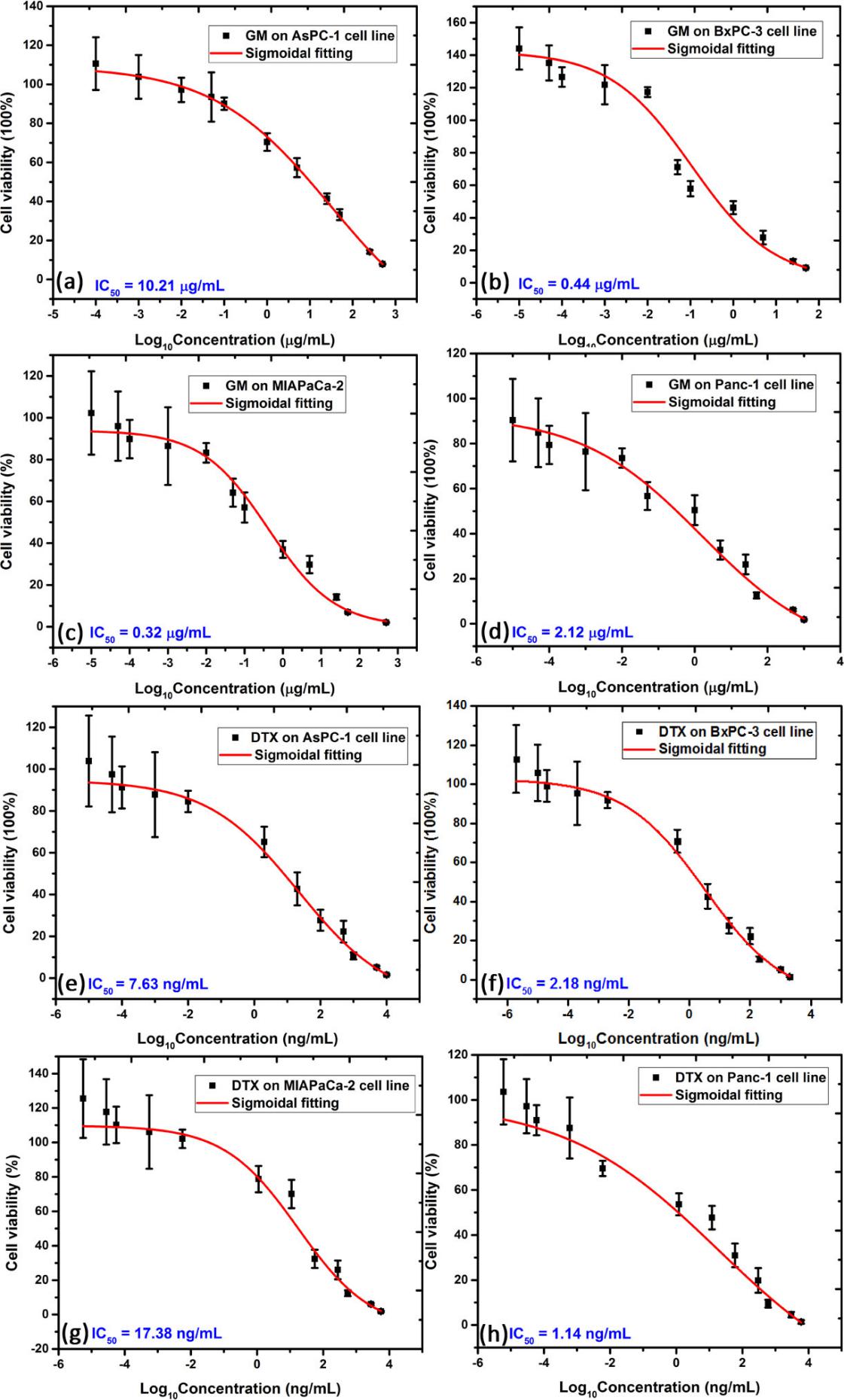


**Figure S3** IC_50_ determination of free GM and DTX treatment groups in 4 different cell lines. The viability of (a) AsPC-1, (b) BxPC-3, (c) MIA PaCa-2 and (d) Panc-1 cells after their exposure to pure GM with different concentrations; (e) AsPC-1, (f) BxPC-3, (g) MIA PaCa-2 and (h) Panc-1 cells after exposure to pure DTX with different concentrations; Data were presented with mean ± standard deviation (SD) (from 6 independent experiments).

**
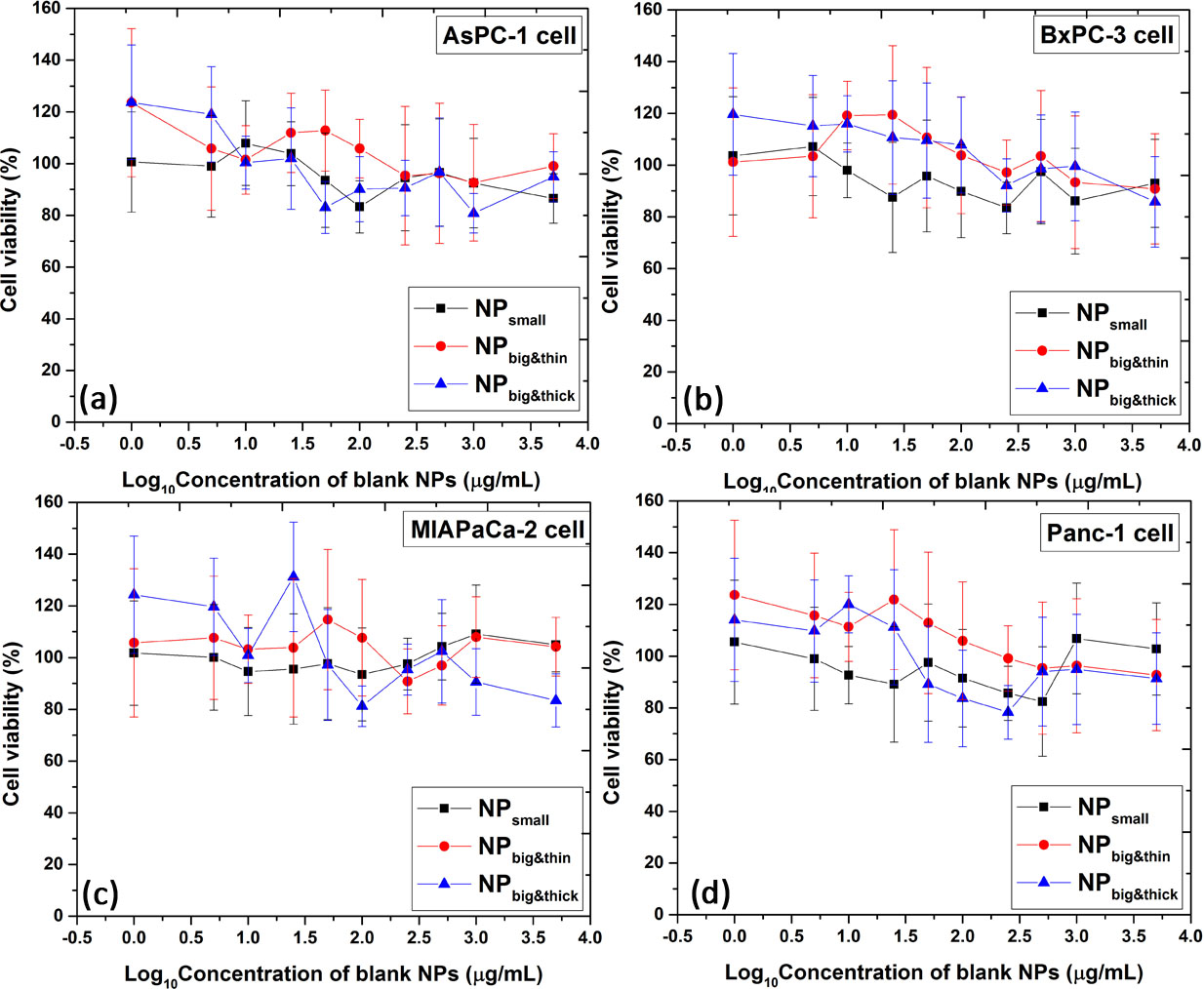
**

**Figure S4** Cytotoxicity evaluation of NP_small_, NP_big&thin_ and NP_big&thick_ in 4 different cells, (a) AsPC-1, (b) BxPC-3, (c) MIA PaCa-2 and (d) Panc-1 cells. Data were presented with mean ± standard deviation (SD) (from 6 independent experiments).


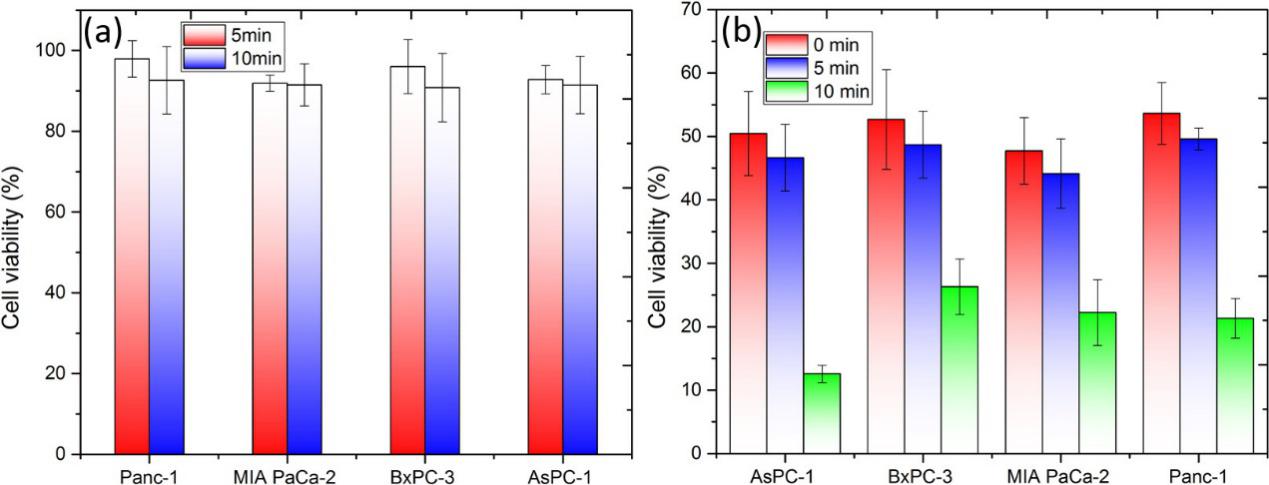


**Figure S5** Cytotoxicity evaluation of Cocktail NPs. (a) Cell viability of 4 different cells after LED light irradiation for 5 or 10 min. (b) Cell viability of 4 different cells at MB concentration of 10 μM and light irradiation duration. Data were presented with mean ± standard deviation (SD) (from 6 independent experiments).


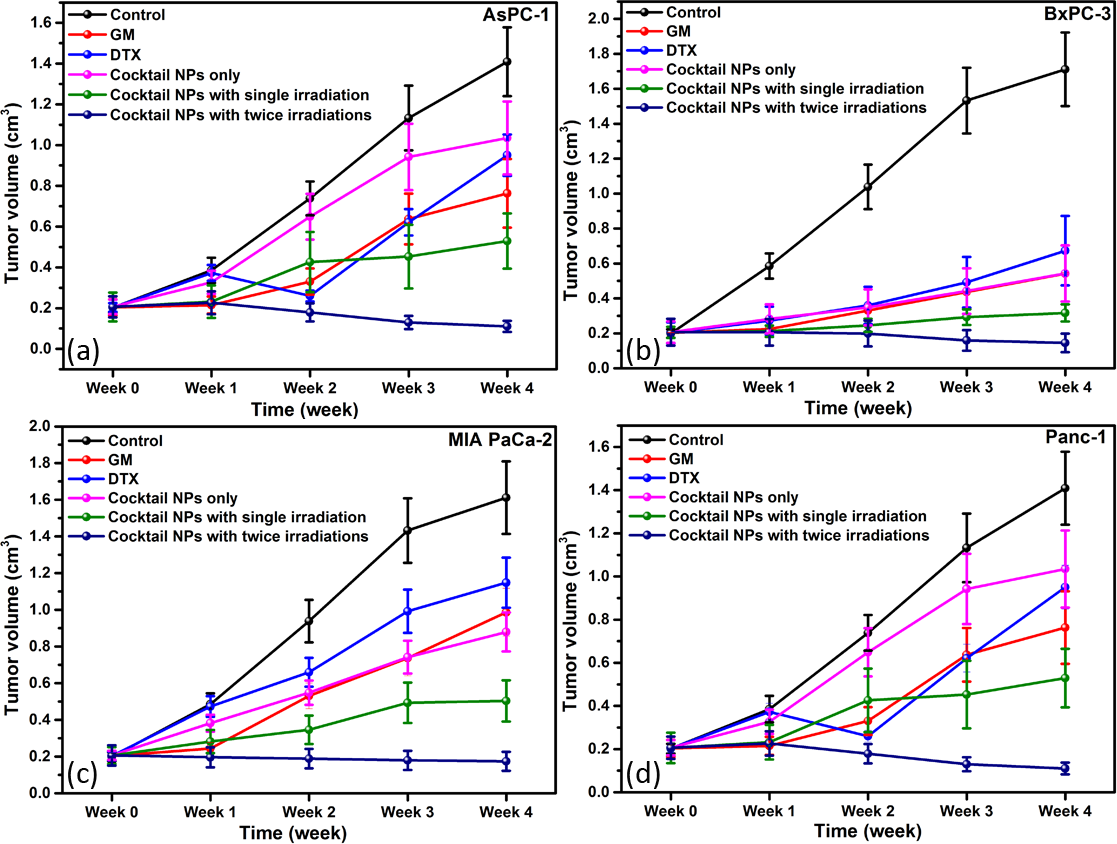


**Figure S6** The tumor size growth curves during the treatment in (a) AsPC-1 cells, (b) BxPC-3 cells, (c) MIA PaCa-2 cells and (d) Panc-1 cells. All experiments were carried out using 5 mice/group, data was shown as Mean±S.D.


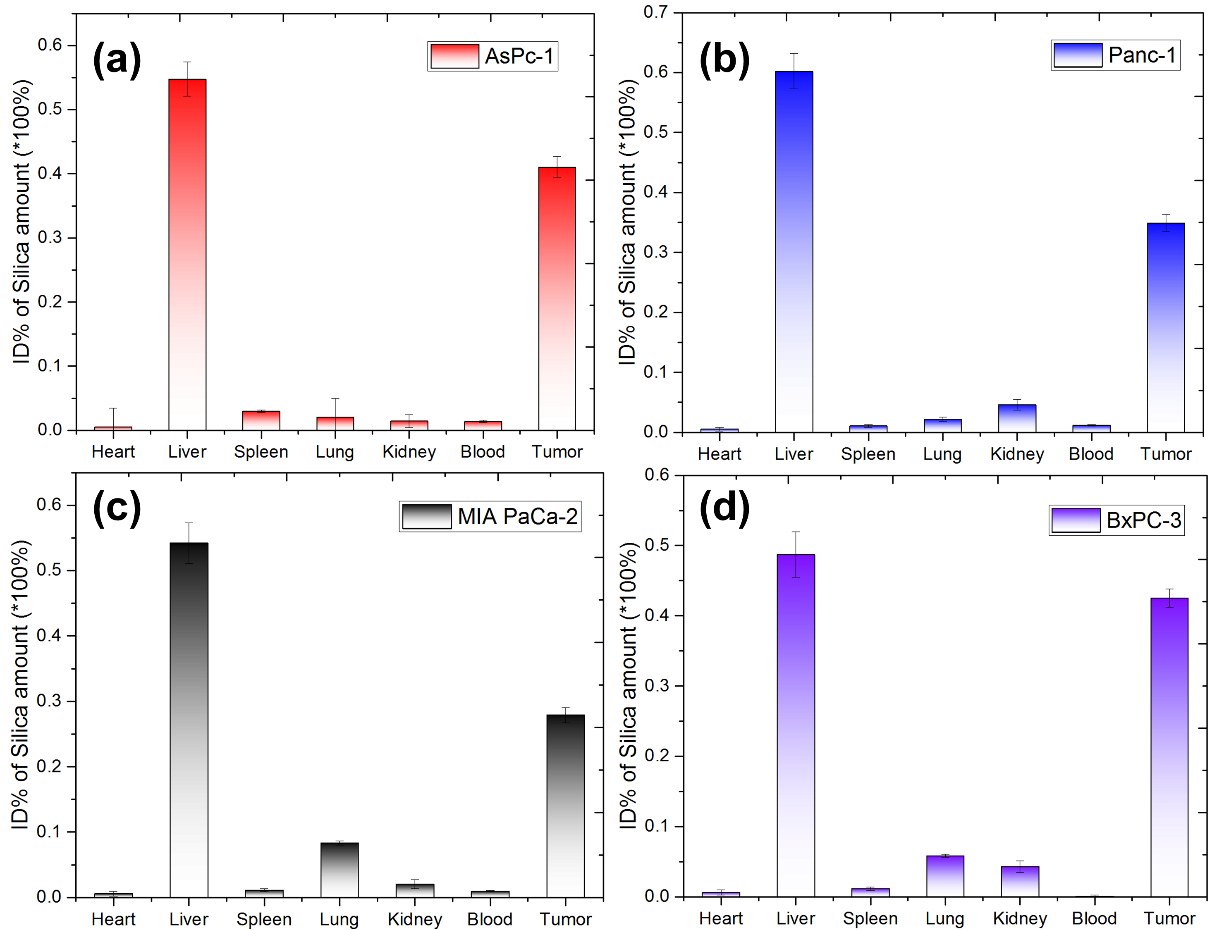


**Figure S7** The bio-distribution of cocktail NPs presented by Silica percentage in (a) AsPC-1, (b) Panc-1, (c) MIA PaCa-2 and (d) BxPC-3 tumors bearing mice. All experiments were carried out using 5 mice/group, data was shown as Mean±S.D.

**Table S1** Statistical analysis of tumor weight treated with Cocktail NPs upon different irradiation protocols in AsPC-1 tumor model. Sidak's multiple comparisons test was applied on each NPs treatment group with free drug, respectively. Significant difference in comparison was mark in yellow frame.

| Sidak's multiple comparisons test on AsPC-1 tumor model | Mean Diff. | 95.00% CI of diff. | Significant ? | Summary | Adjusted P Value |
| --- | --- | --- | --- | --- | --- |
| GM vs. Cocktail NPs only | **-0.264** | **-0.6489~0.1216** | **No** | **ns** | **0.3255** |
| GM vs. Cocktail NPs with single irradiation at 6 hrs | **0.272** | **-0.1134~0.6571** | **No** | **ns** | **0.2927** |
| GM vs. Cocktail NPs with double irradiations at 6 hrs and 18 hrs | **0.401** | **0.01524~0.7858** | **Yes** | ***** | **0.0382** |
| DTX vs. Cocktail NPs only | **-0.103** | **-0.4885~0.282** | **No** | **ns** | **0.9741** |
| DTX vs. Cocktail NPs with single irradiation at 6 hrs | **0.432** | **0.04695~0.8175** | **Yes** | ***** | **0.0214** |
| DTX vs. Cocktail NPs with double irradiations at 6 hrs and 18 hrs | **0.561** | **0.1756~0.9461** | **Yes** | ****** | **0.0017** |

**Table S2** Statistical analysis of tumor weight treated with Cocktail NPs upon different irradiation protocols in BxPC-3 tumor model. Sidak's multiple comparisons test was applied on each NPs treatment group with free drug, respectively. Significant difference in comparison was mark in yellow frame.

| Sidak's multiple comparisons test on BxPC-3 tumor model | Mean Diff. | 95.00% CI of diff. | Significant ? | Summary | Adjusted P Value |
| --- | --- | --- | --- | --- | --- |
| GM vs. Cocktail NPs only | **-0.001** | **-0.5242~0.5222** | **No** | **ns** | **>0.9999** |
| GM vs. Cocktail NPs with single irradiation at 6 hrs | **0.225** | **-0.2981~0.7482** | **No** | **ns** | **0.7999** |
| GM vs. Cocktail NPs with double irradiations at 6 hrs and 18 hrs | **0.397** | **-0.1264~ 0.92** | **No** | **ns** | **0.222** |
| DTX vs. Cocktail NPs only | **0.130** | **-0.3932~0.6532** | **No** | **ns** | **0.9823** |
| DTX vs. Cocktail NPs with single irradiation at 6 hrs | **0.356** | **-0.1671~0.8793** | **No** | **ns** | **0.3311** |
| DTX vs. Cocktail NPs with double irradiations at 6 hrs and 18 hrs | **0.528** | **0.00467~1.051** | **Yes** | ***** | **0.0471** |

**Table S3** Statistical analysis of tumor weight treated with Cocktail NPs upon different irradiation protocols in MIA PaCa-2 tumor model. Sidak's multiple comparisons test was applied on each NPs treatment group with free drug, respectively. Significant difference in comparison was mark in yellow frame.

| Sidak's multiple comparisons test on MIA PaCa-2 tumor model | Mean Diff. | | 95.00% CI of diff. | Significant ? | Summary | Adjusted P Value |
| --- | --- | --- | --- | --- | --- | --- |
| GM vs. Cocktail NPs only | | **0.107** | **-0.5484~ 0.7626** | **No** | **ns** | **0.9981** |
| GM vs. Cocktail NPs with single irradiation at 6 hrs | | **0.483** | **-0.1725~ 1.138** | **No** | **ns** | **0.2493** |
| GM vs. Cocktail NPs with double irradiations at 6 hrs and 18 hrs | | **0.712** | **0.05681~1.368** | **Yes** | ***** | **0.0275** |
| DTX vs. Cocktail NPs only | | **0.268** | **-0.3867~ 0.9242** | **No** | **ns** | **0.8322** |
| DTX vs. Cocktail NPs with single irradiation at 6 hrs | | **0.644** | **-0.01084~ 1.3** | **No** | **ns** | **0.0559** |
| DTX vs. Cocktail NPs with double irradiations at 6 hrs and 18 hrs | | **0.873** | **0.2185~1.529** | **Yes** | ****** | **0.0045** |

**Table S4** Statistical analysis of tumor weight treated with Cocktail NPs upon different irradiation protocols in Panc-1 tumor model. Sidak's multiple comparisons test was applied on each NPs treatment group with free drug, respectively. Significant difference in comparison was mark in yellow frame.

| Sidak's multiple comparisons test on Panc-1 tumor model | Mean Diff. | | 95.00% CI of diff. | Significant ? | Summary | Adjusted P Value |
| --- | --- | --- | --- | --- | --- | --- |
| GM vs. Cocktail NPs only | | **-0.066** | **-0.3016~0.1696** | **No** | **ns** | **0.968** |
| GM vs. Cocktail NPs with single irradiation at 6 hrs | | **0.321** | **0.0851~0.556** | **Yes** | ****** | **0.0036** |
| GM vs. Cocktail NPs with double irradiations at 6 hrs and 18 hrs | | **0.505** | **0.2696~0.7409** | **Yes** | ******** | **<0.0001** |
| DTX vs. Cocktail NPs only | | **0.152** | **-0.0836~0.3877** | **No** | **ns** | **0.3907** |
| DTX vs. Cocktail NPs with single irradiation at 6 hrs | | **0.539** | **0.3031~0.7744** | **Yes** | ******** | **<0.0001** |
| DTX vs. Cocktail NPs with double irradiations at 6 hrs and 18 hrs | | **0.723** | **0.4876~0.9589** | **Yes** | ******** | **<0.0001** |
